# A bibliometric and visualization analysis of the role of traditional Chinese medicine in cancer immunotherapy

**DOI:** 10.3389/fimmu.2025.1499026

**Published:** 2025-02-14

**Authors:** Yixiao Lian, Jie Sun, Lin Yang, Weidong Yu

**Affiliations:** ^1^ Department of Library, Peking University People’s Hospital, Beijing, China; ^2^ Department of Central Laboratory and Institute of Clinical Molecular Biology, Peking University People’s Hospital, Beijing, China

**Keywords:** VOSviewer, CiteSpace, Bibliometrix, traditional Chinese medicine, immunotherapy, cancer

## Abstract

**Objective:**

Traditional Chinese medicine (TCM) is used as a complementary treatment for patients with cancer, especially in immunotherapy. Although extensive clinical and basic research has been conducted on TCM in cancer immunotherapy, a comprehensive bibliometric analysis of this field has not yet been performed. This study aimed to investigate the progress and status of TCM, and the research focused on cancer immunotherapy.

**Methods:**

We collected 1,657 articles on TCM in cancer immunotherapy from 1994 to 2024 from the Web of Science Core Collection database. VOSviewer, CiteSpace, and the Bibliometrix R package were used to analyze countries, institutions, journals, authors, references, and keywords to predict future trends in cancer immunotherapy with TCM.

**Results:**

The publication rate of TCM in cancer immunotherapy research steadily increased from 1994 to 2018, with a swift growth from 2018 to 2023. China and TCM universities have achieved the most research advancements in this field. The most studied types of cancer are liver, lung, and colorectal cancers. However, few studies exist on upper respiratory tract tumors, cervical cancer, and melanoma, which deserve more attention. The study trend has gradually shifted from *in vivo* and *in vitro* models to clinical efficacy. Simultaneously, the focus of research transitioned from compound TCM preparations or classes of ingredients to specific pharmacodynamic ingredients, and the corresponding targets transitioned from cytokines to immune checkpoints. In general, molecular docking combined with multi-omics analysis is a popular and trending research method in TCM for cancer immunotherapy, helping researchers understand the mechanisms of TCM in cancer immunotherapy more comprehensively and accurately. By analyzing the literature, it is evident that TCM-based immunotherapy should contribute to effective maintenance or adjuvant therapy throughout the entire course of cancer rather than only in the late stages.

**Conclusion:**

This study comprehensively summarized and identified research frontiers providing a reference for promoting the development of TCM immunotherapy preparations and guiding clinical practice. Consequently, more patients with cancer can benefit from immunotherapy.

## 1 Introduction

Cancer is a chronic condition that profoundly impacts human health and stands as the foremost cause of mortality globally. It presents a major barrier to improving life expectancy across populations (1). In 2020, approximately 19.3 million new cases of cancer were estimated, with approximately 10 million deaths attributable to the disease worldwide. Projections indicate that by 2040, the global cancer burden is expected to escalate to about 28.4 million cases, representing a substantial 47% increase compared to 2020 figures (2). Currently, cancer treatments mainly comprise surgery, chemotherapy, targeted therapy, radiotherapy, and immunotherapy (3–6). Among these modalities, chemotherapy remains the most commonly employed treatment method for cancer. Despite advancements in chemotherapeutic agents, the efficacy of chemotherapy is often limited by drug resistance and disease recurrence (7, 8). These challenges can lead to diminished survival rates for patients with cancer. The overarching goal of cancer treatment should not only be to extend the survival of patients but also to enhance their quality of life throughout the illness (9). Consequently, cancer prevention and treatment pose significant challenges that necessitate ongoing research and innovative solutions globally.

Immunotherapy is a rapidly developing field that has achieved significant success in the development of cancer therapies. Cancer immunotherapies, including checkpoint inhibitors and adoptive cell therapy, are considered innovative approaches alongside traditional treatments, including surgery, cytotoxic chemotherapy, radiation, and targeted therapy (10). Briefly, immunotherapy is a means of reshaping the immune system to reactivate the anti-tumor response and prevent tumor escape. Early approaches to immunotherapy in cancer targeted cytokines. In the 1990s, the FDA approved interleukin-2 (IL-2) for the treatment of renal cell carcinoma and metastatic melanoma (11). Currently, treatments with inhibitors targeting immune checkpoints related to T cells, such as programmed cell death protein 1/programmed death-ligand 1 (PD-1/PD-L1) and cytotoxic T-lymphocyte-associated protein 4, have been used for various cancers, including melanoma, non-small-cell lung cancer, and head and neck cancer (12–14). Therefore, minimizing tumor immunosuppression could be an important strategy for cancer therapy.

The term “immunity” in TCM was first seen in the Mian Yi Lei Fang (or “Immune Formularies”) in the Ming Dynasty. When the human body has balanced yin and yang and normal immune function, it can fight against and dispel pathogenic qi, and thus pathogenic qi will not cause harm to the human body. There is no “immunotherapy“ in Chinese medicine, and the understanding of immunity in Chinese medicine is mainly reflected in the word “positive qi”. Therefore, a healthy or sick body depends mainly on the rise and fall of vital qi (15). Starting from classic theories such as “harmonizing yin and yang” and “supporting the vital qi to dispel pathogenic qi”, TCM-based treatment of malignant tumors is characterized by its holistic and balanced approaches. Modern research has confirmed that TCM can regulate antitumor immunity. By regulating the TME and reshaping the immunosuppressive cells in the TME, TCM can prevent and treat tumor metastasis/recurrence and enhance the body’s immune response (16), ultimately enabling patients to live with tumors.

Traditional Chinese medicines (TCMs) may exert therapeutic effects by directly targeting cancer cells, reducing the side effects of anti-tumor drugs, or controlling tumor growth and metastasis by enhancing anti-tumor immunity. The concept of “strengthening resistance to eliminate pathogenic factors” in TCM aligns with cancer immunotherapy (17, 18). Modern pharmacological research has shown that TCM can improve the efficacy of tumor immunotherapy by inhibiting the overexpression of immune checkpoint molecules, directly or indirectly affecting the tumor microenvironment (TME). According to previous studies, the role of TCM in tumor immunotherapy is mainly associated with the positive regulation of natural killer (NK) cells, CD8/CD4 T cells, dendritic cells, M2 macrophages, IL-2, tumor necrosis factor-α (TNF-α), interferon-γ (IFN-γ), the negative regulation of regulatory T cells (Tregs), myeloid-derived suppressor cells (MDSCs), cancer-associated fibroblasts, PD-1/PD-L1, transforming growth factor-β, and TNF-β. The combination of TCM and immune regulation stimulation may be a key factor in helping patients with non-inflammatory or “cold” tumors benefit from immunotherapy (19, 20).

Pritchard and Hawkins defined bibliometry as “the application of mathematical and statistical methods to books and other media of communication” and “the quantitative analysis of the bibliographic features of a body of literature”, respectively (21). In contemporary academia, researchers spanning various disciplines are increasingly employing bibliometric methods to gain rapid insights into emerging research frontiers and focal points within specific fields. However, despite the growing interest in TCM in cancer immunotherapy, no comprehensive bibliometric analysis has been conducted on this intersection. To address this gap, the present study used R software alongside VOSviewer and CiteSpace to extensively analyze the literature concerning TCM in the context of cancer immunotherapy. The primary objective was to investigate the evolution and emerging research trends in the application of TCM in cancer immunotherapy between 1994 and 2024. By fostering a deeper understanding of the current landscape and future potential of TCM in anti-tumor immunotherapy, this research seeks to contribute to the sustainable advancement of this field, ultimately enhancing the efficacy and application of TCM in cancer treatment modalities.

## 2 Materials and methods

### 2.1 Data source and literature search strategy

Web of Science (WoS) is one of the most reliable databases with multidisciplinary coverage, including over 12,000 international academic journals, and was selected as the primary database for this study. In addition, WoS can track more comprehensive citations of publications for better citation analyses, especially older citations, but PubMed lack this part of data, and WoS is considered more user-friendly for bibliometric analysis compared to other databases, such as PubMed and Scopus (22). All literature published in the WoS Core Collection (WoSCC) was searched and exported on April 24, 2024. We used the following search strategy: [ALL = (immune therapy OR immunotherapy OR immune OR immunity OR immunotherapies) AND ALL = (cancer* OR tumor* OR carcinoma* OR neoplasms *) AND ALL = (traditional Chinese medicine OR TCM OR Chinese medicine OR Chinese herbal medicine* OR natural ingredients OR natural compounds OR natural products) AND Document Type = (Article AND Review) AND Language = English]. Full records and cited references were extracted from the retrieved publications and saved in plain text format for further analysis. Subsequently, the two authors(YL and JS) excluded articles that was not relevant to the topic and keywords in the “Address” field. Divergent viewpoints would be resolved through discussion or a third party (LY and WY).

### 2.2 Software for visualization analysis

This study used the Bibliometrix package in R software (version 4.4.0), VOSviewer (version 1.6.17), and CiteSpace (version 6.3. R2 Advanced) for bibliometric analysis and the creation of scientific knowledge maps (23–25).

**Figure 1:**
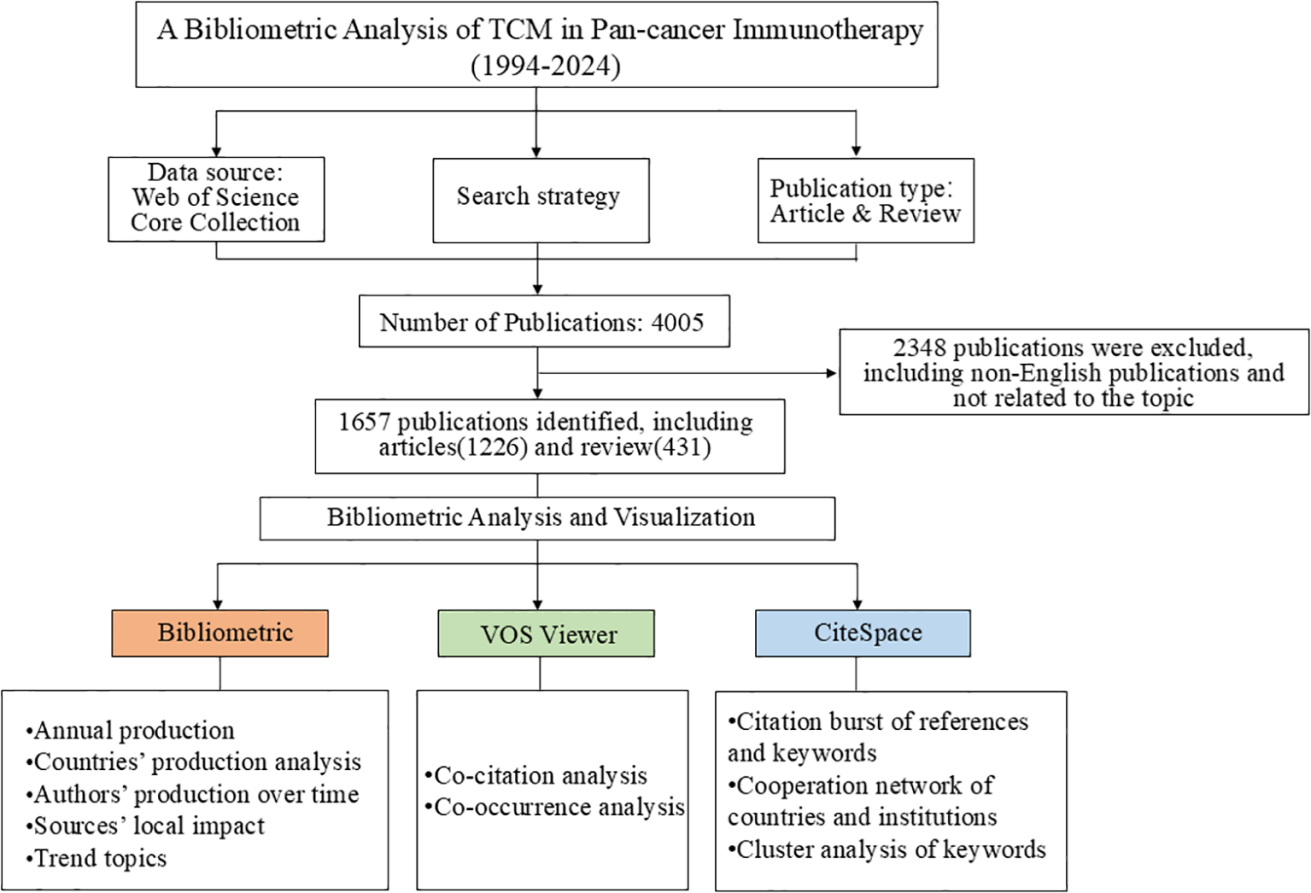
Flowchart depicting the article selection process.

The Bibliometrix R package was mainly used for analyzing annual production, country-wise production, authors’ contributions over time, local impacts of sources based on the H index, and trending topics (**Figure 1**). VOSviewer software was used to perform co-citation and co-occurrence analyses, with co-citation analysis focusing mainly on cited journals and references. CiteSpace software was used to analyze citation bursts in references and keywords, visualize the cooperation network of countries and institutions, and perform cluster analysis of keywords. The relative parameter settings of VOSviwer main about the minimum number of documents/citations/times. In details,1) for co-authorship analysis, the minimum number of documents of an author was defined as more than 5; 2) for co-citation analysis, the minimum number of citations of a source and cited reference were defined as more than 30 and 15 respectively; 3) for co-occurrence analysis, the minimum number of occurrences of a keyword was defined as more than 15. The parameters of Citespace were set as follows: Timespan:1994-2024, years per slice:1, selection criteria (g-index:K = 25), link retaining factor (LRF = 3), look back years (LBY = 5), e for top N(e = 1), and minimum duration (MD = 2 for keywords; MD = 2 for references).

## 3 Results

### 3.1 General landscapes of global publications

**Figure 2:**
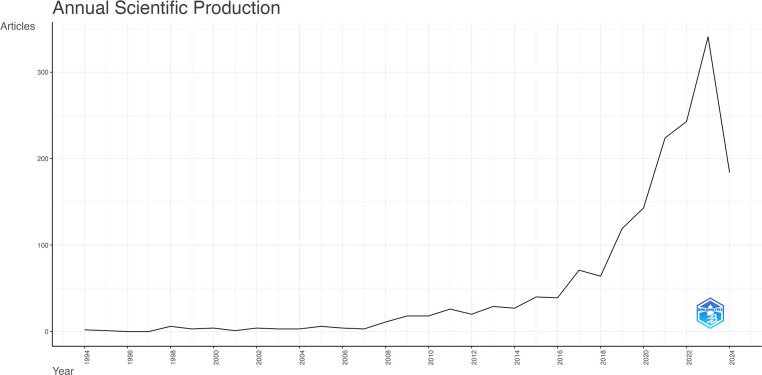
The annual number of publications related to traditional Chinese medicine in cancer immunotherapy from 1994 to 2024.

Based on the search strategy, 4,005 publications were collected from WoSCC without duplicates. Finally, 1,657 publications were identified after excluding those unrelated to the topic. The trend in global publications on TCM for cancer immunotherapy has increased steadily each year (**Figure 2**). From 1994 to 2018, the number of relevant publications increased gradually. From 2018 to 2023, the number of publications increased rapidly, reaching 341 in 2023.

### 3.2 Contribution of publications volume and collaboration in countries and regions

**Figure 3:**
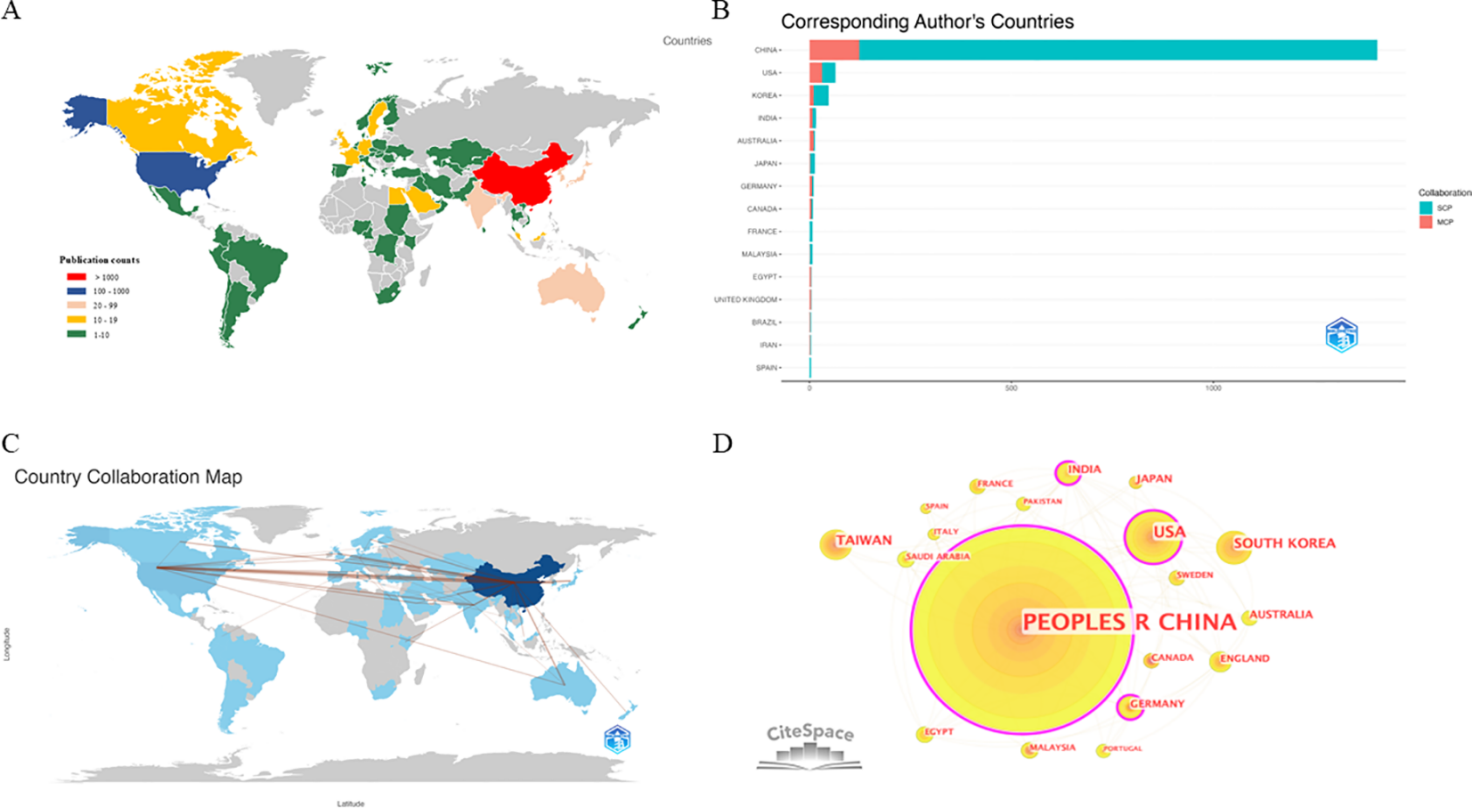
Global trends and countries/regions contributing to the research field regarding TCM in cancer immunotherapy from 1994 to 2024. **(A)** A map of country contribution based on the publication output. **(B)** The top 15 countries of total number of publications. **(C)** A visual map for country collaboration. **(D)** Country/regional collaboration analysis. The nodes represent countries/regions, and the number of publications grows proportionally to the size of the nodes. The lines between the nodes represent the cooperation relationship, and the thickness of the connecting lines represents the strength of their cooperation, the closer the cooperation, the thicker the connecting lines. The nodes with the outermost purple circles have higher centrality (Centrality: CHINA = 0.69; USA = 0.29; INDIA = 0.26;GERMANY = 0.23). Created with CiteSpace.

The national publication output analysis showed that 64 countries/regions published articles in relevant fields. The distribution of publications by country is shown in **Figure 3A**. China (n = 1,405) was the most productive country, accounting for 84.8% of the total publications, followed by the United States (n = 64, 3.9%), the Republic of Korea (n = 47, 2.8%), India (n = 16, 1.0%), Australia (n = 13, 0.8%), and Japan (n = 13, 0.8%) (**Figure 3B**, **Table 1**). The results demonstrated that compared to other countries and regions, China had a much larger number of articles in this field, most of which were SCP, accounting for 91.2% of the total publications from China. Interestingly, Australia had the highest MCP ratio (0.769) compared to China (0.088).

**Table 1:** The top 15 productive countries/regions related to TCM in cancer immunotherapy.

Rank	Country/Region	Article count	SCP	MCP	Percentage	Total cites
1	CHINA	1405	1282	123	84.80%	24306
2	USA	64	33	31	3.90%	2943
3	KOREA	47	37	10	2.80%	1107
4	INDIA	16	9	7	1.00%	607
5	AUSTRALIA	13	3	10	0.80%	144
6	JAPAN	13	11	2	0.80%	428
7	GERMANY	10	4	6	0.60%	479
8	CANADA	8	3	5	0.50%	632
9	FRANCE	7	7	0	0.40%	129
10	MALAYSIA	7	6	1	0.40%	236
11	EGYPT	5	1	4	0.30%	74
12	UNITED KINGDOM	5	1	4	0.30%	175
13	BRAZIL	4	2	2	0.20%	106
14	IRAN	4	2	2	0.20%	92
15	SPAIN	4	4	0	0.20%	579

**Figure 3C** shows a further analysis of the collaboration among countries and regions. The most frequent collaboration was between China and the United States (frequency = 70), followed by China and Australia (frequency = 16), China and the United Kingdom (frequency = 14), and China and Japan (frequency = 13). Among the top 10 collaborations, except for two relationships between the United States and India, and the United States and Italy, all international collaborations involved China.

A global collaboration network analysis was also conducted using CiteSpace. China exhibited the highest volume output and worked closely with other countries, indicating its strongest international collaborations (**Figure 3D**). The size of the circle represents the collaboration strength, whereas the color outside the circle represents the collaboration distribution. The four countries with the highest degrees of centrality were China (centrality = 0.69), the United States (centrality = 0.29), India (centrality = 0.26), and Brazil (centrality = 0.23), indicating that these four countries held leading positions in this field.

### 3.3 Analysis of institutions and authors

**Figure 4:**
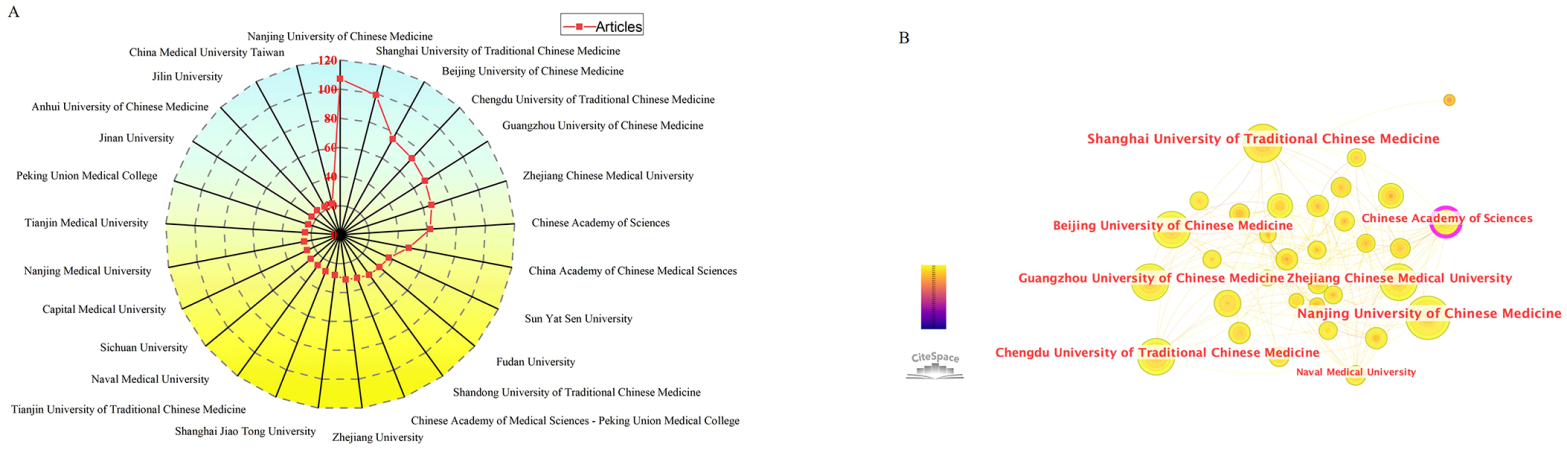
Institutions and their cooperative relationship in the field regarding TCM in cancer immunotherapy. **(A)** The top 25 institutions with most publications related to TCM in cancer immunotherapy. **(B)** Institutions collaboration analysis. Created with CiteSpace.

A total of 1356 institutions conducted research on TCM in cancer immunotherapy. The top 25 most productive institutions were all from China (**Figure 4A**). Nanjing University of Chinese Medicine had the most publications (n = 107), followed by Shanghai University of Traditional Chinese Medicine (n = 99) and Beijing University of Chinese Medicine (n = 75). To further investigate collaborations between institutions, we conducted a co-authorship analysis using CiteSpace (**Figure 4B**). Interestingly, the Chinese Academy of Sciences published 62 papers, ranking 7th in terms of the number of articles, but had the strongest influence in this research field (centrality = 0.22). The Shanghai University of Traditional Chinese Medicine ranked second (centrality = 0.10). These findings suggest that Chinese medical institutions have published the most articles in this research area. Furthermore, the collaboration between these institutions should be strengthened.

**Figure 5:**
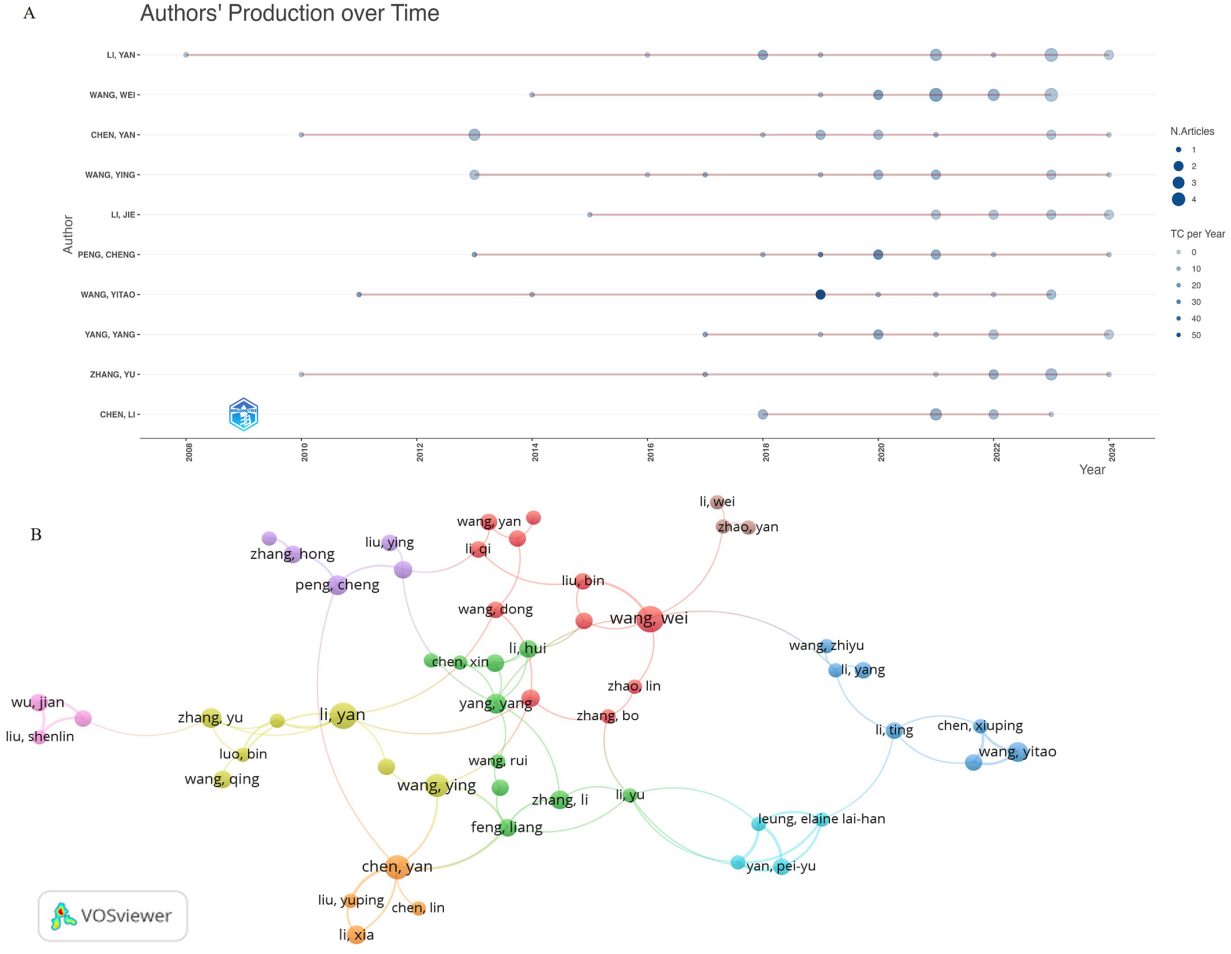
Visualization of active authors analysis. **(A)** Timeline distribution of the top 10 most productive authors. **(B)** Network map showing cooperation among authors. Created with VOSviewer.

The most productive authors were Wang Wei and Li Yan, each with 15 publications, while Wang Yitao was the most-cited author, with 688 citations (**Table 2**). **Figure 5A** shows the authors’ production over time. The size of the circle represented the number of publications, while the color represented the total number of citations per year. Notably, Wang Yitao had the highest number of citations in 2019, despite having only two publications. **Figure 5B** illustrates the co-authorship clustering network among researchers, revealing their collaborative relationships. Fifty-four authors were classified into nine clusters centered around the most productive authors (**Table 2**).

**Table 2:** The top 10 authors with the most publications on TCM in cancer immunotherapy treatment.

Rank	High Published Authors	County	Article counts	Total cites
1	WANG, YITAO	China	9	688
2	PENG, CHENG	China	9	596
3	WANG, WEI	China	15	272
4	CHEN, YAN	China	13	263
5	WANG, YING	China	12	215
6	YANG, YANG	China	9	212
7	LI, YAN	China	15	203
8	ZHANG, YU	China	9	128
9	CHEN, LI	China	8	117
10	LI, JIE	China	9	65

### 3.4 Journals and co-cited journals

**Figure 6:**
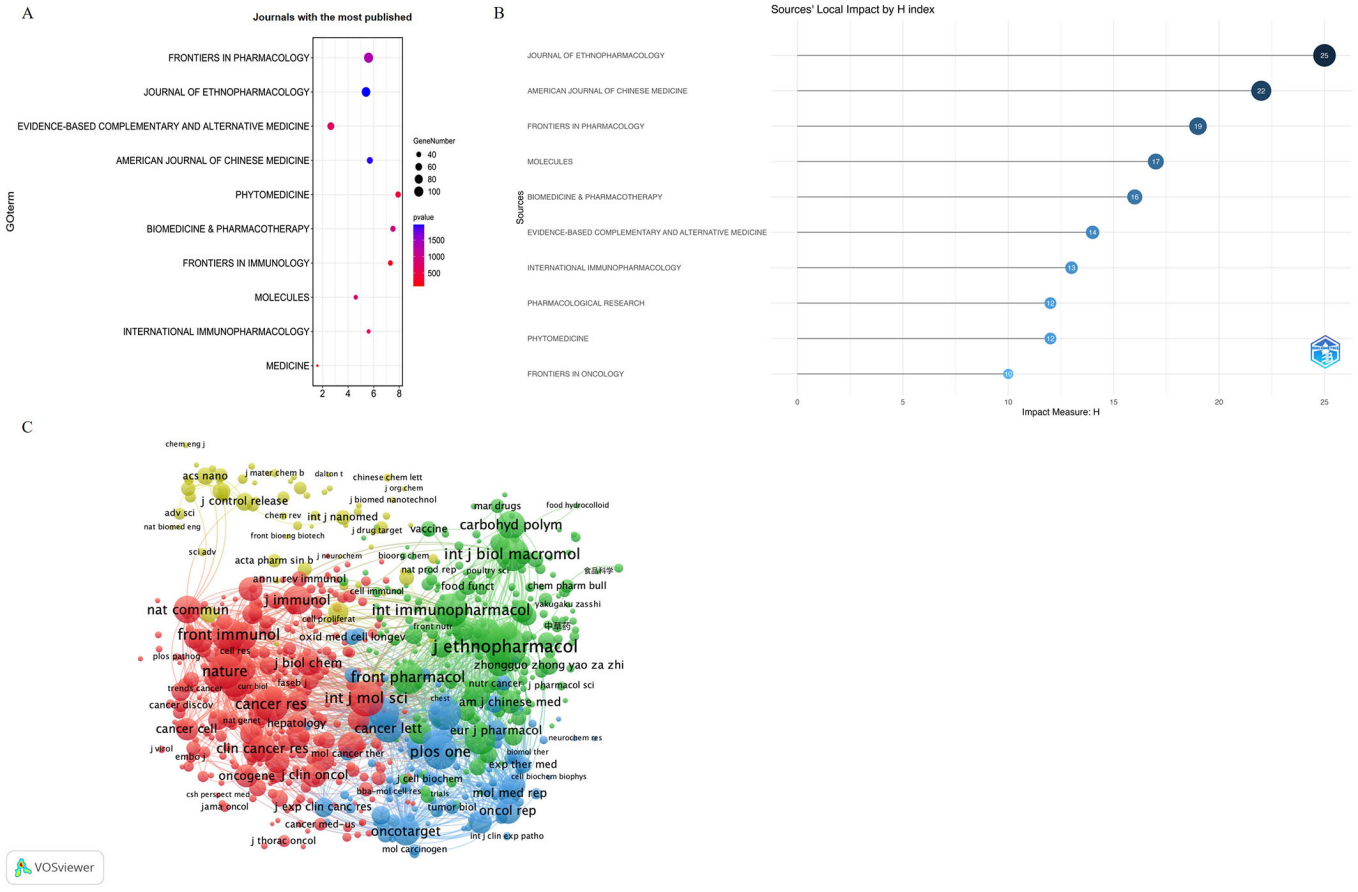
Analysis of journal in the field of regarding TCM in cancer immunotherapy. **(A)** Journal with the largest number of articles published. **(B)** Timeline distribution of the top 10 most productive Journals. **(C)** Co-cited journals involved in TCM in cancer immunotherapy. Created with VOSviewer.

This study included 1,657 articles published in 464 journals. **Figure 6A** and **Table 3** list the top 10 journals ranked by publication quantity and their latest 2023 impact factors (IF) (26). These include *Frontiers in Pharmacology* (n = 100, IF = 4.4), *Journal of Ethnopharmacology* (n = 91, IF = 4.8), *Evidence-Based Complementary and Alternative Medicine* (n = 61, IF = 2.65), *American Journal of Chinese Medicine* (n = 51, IF = 4.8), and Phytomedicine (n = 45, IF = 6.7). We used R-Bibliometrix to measure journal impact based on the H-index. The *Journal of Ethnopharmacology* had the highest H-index (**Figure 6B**) (27). Notably, the limited presence of publications in top-tier journals on the achievements of TCM in cancer immunotherapy suggests the need to improve the quality of research in this field.

**Table 3:** The top 10 productive journals related to TCM in cancer immunotherapy treatment.

Rank	Journal	Article counts	Percentage%	IF	CITES
1	FRONTIERS IN PHARMACOLOGY	100	6.0%	4.4	1351
2	JOURNAL OF ETHNOPHARMACOLOGY	91	5.5%	4.8	1968
3^*^	EVIDENCE-BASED COMPLEMENTARY AND ALTERNATIVE MEDICINE	61	3.7%	2.65	703
4	AMERICAN JOURNAL OF CHINESE MEDICINE	51	3.1%	4.8	1935
5	PHYTOMEDICINE	45	2.7%	6.7	426
6	BIOMEDICINE & PHARMACOTHERAPY	43	2.6%	6.9	982
7	FRONTIERS IN IMMUNOLOGY	39	2.4%	5.7	220
8	MOLECULES	35	2.1%	4.2	863
9	INTERNATIONAL IMMUNOPHARMACOLOGY	33	2.0%	4.8	644
10	MEDICINE	29	1.8%	1.3	103

*The journal was removed from the SCIE journal catalog in 2022.

The source titles of the co-citation analysis were analyzed using VOSviewer, and journals with at least 30 citations were included. Five hundred eighty-one journals were identified based on their total link strength (**Figure 6C**). The top five journals with the highest total link strength were the *Journal of Ethnopharmacology* (total link strength = 123818), *International Journal of Molecular Sciences* (total link strength = 118496), *PLOS One* (total link strength = 110081), *Biomedicine & Pharmacotherapy* (total link strength = 108686), and *Oncotarget (*total link strength = 101613).

### 3.5 Co-cited references and reference bursts

The top 10 most-cited publications were listed in **Table 4**. The publication titled “Resveratrol as an Anti-Inflammatory and Anti-Aging Agent: Mechanisms and Clinical Implications” received 558 citations, followed by “The Scientific Rediscovery of an Ancient Chinese Herbal Medicine: *Cordyceps sinensis* Part I”, with 383 citations, and “Advances in Saponin-Based Adjuvants,” with 324 citations. Highly cited papers were indicators of the Essential Science Indicators (ESI) database used to explore the research frontier (27). Among the 10 publications, four were highly cited papers that provide significant guidance in the field.

**Table 4:** The top 10 documents with the citations in the field of TCM in cancer immunotherapy treatment.

Rank	Article Title	Journal	IF	Publication Year	Times Cited	Highly Cited Status
1	Resveratrol as an anti-inflammatory and anti-aging agent: Mechanisms and clinical implications	MOLECULAR NUTRITION & FOOD RESEARCH	4.5	2005	558	
2	The scientific rediscovery of an ancient Chinese herbal medicine: Cordyceps sinensis Part I	JOURNAL OF ALTERNATIVE AND COMPLEMENTARY MEDICINE	2.3	1998	383	
3	Advances in saponin-based adjuvants	VACCINE	4.5	2009	324	
4	Use of complementary/alternative medicine by breast cancer survivors in Ontario: Prevalence and perceptions	JOURNAL OF CLINICAL ONCOLOGY	42.1	2000	306	
5	The advantages of using traditional Chinese medicine as an adjunctive therapy in the whole course of cancer treatment instead of only terminal stage of cancer	BIOSCIENCE TRENDS	5.7	2015	302	Y
6	Chinese herbal medicines as adjuvant treatment during chemo- or radio-therapy for cancer	BIOSCIENCE TRENDS	5.7	2010	291	
7	Naturally occurring anti-cancer compounds: shining from Chinese herbal medicine	CHINESE MEDICINE	5.3	2019	255	Y
8	Astragalus membranaceus: A Review of its Protection Against Inflammation and Gastrointestinal Cancers	AMERICAN JOURNAL OF CHINESE MEDICINE	4.8	2016	227	Y
9	A review on phytochemistry and pharmacological activities of the processed lateral root of Aconitum carmichaelii Debeaux	JOURNAL OF ETHNOPHARMACOLOGY	4.8	2015	223	Y
10	Current Evaluation of the Millennium Phytomedicine- Ginseng (II): Collected Chemical Entities, Modern Pharmacology, and Clinical Applications Emanated from Traditional Chinese Medicine	CURRENT MEDICINAL CHEMISTRY	3.5	2009	221	

**Figure 7:**
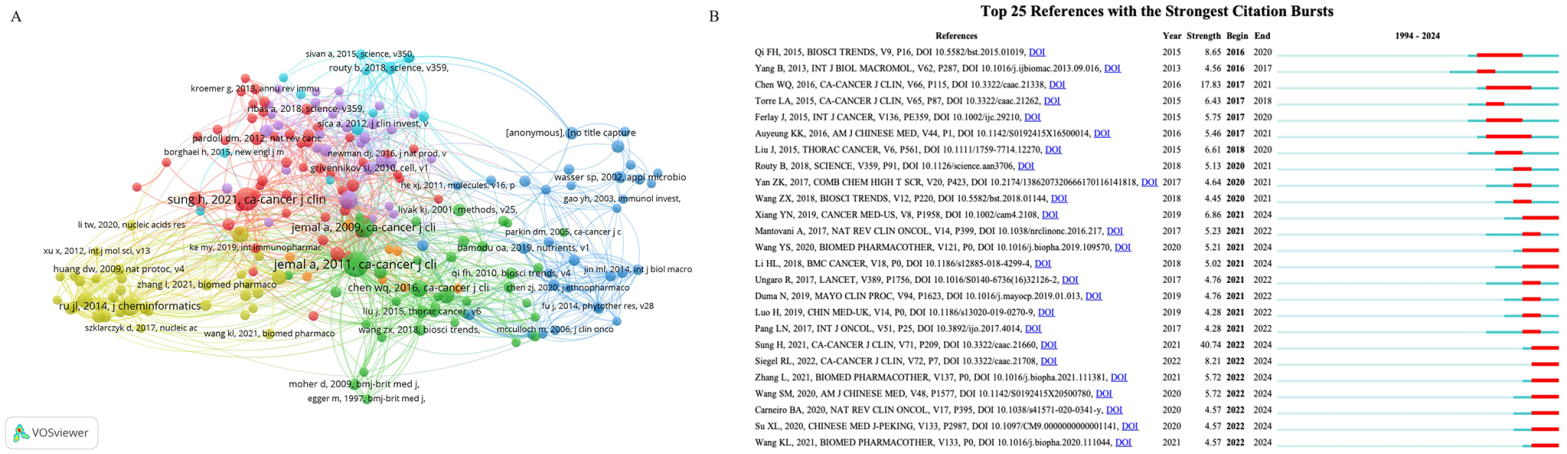
Analysis results of co-cited references. **(A)** The co-citation network of references. **(B)** Top 25 references with the strongest citation bursts on TCM in cancer immunotherapy research. Created with VOSviewer.

Co-cited references were analyzed using VOSviewer to identify the most influential publications (**Figure 7A**). Additionally, CiteSpace was used to identify the most significant citation bursts of TCM in cancer immunotherapy. This analysis yielded 42 references with the strongest citation bursts, 25 of which were presented in **Figure 7B**. Among them, “Global Cancer Statistics 2018: GLOBOCAN Estimates of Incidence and Mortality Worldwide for 36 Cancers in 185 Countries” (strength: 40.74), “Cancer Statistics in China, 2015” (strength: 17.83), and “The Advantages of Using Traditional Chinese Medicine as an Adjunctive Therapy in the Whole Course of Cancer Treatment Instead of Only Terminal Stage of Cancer” (strength: 8.65) were the top three references with the most influential citation bursts.

### 3.6 Analysis of keywords and hotspots

**Figure 8:**
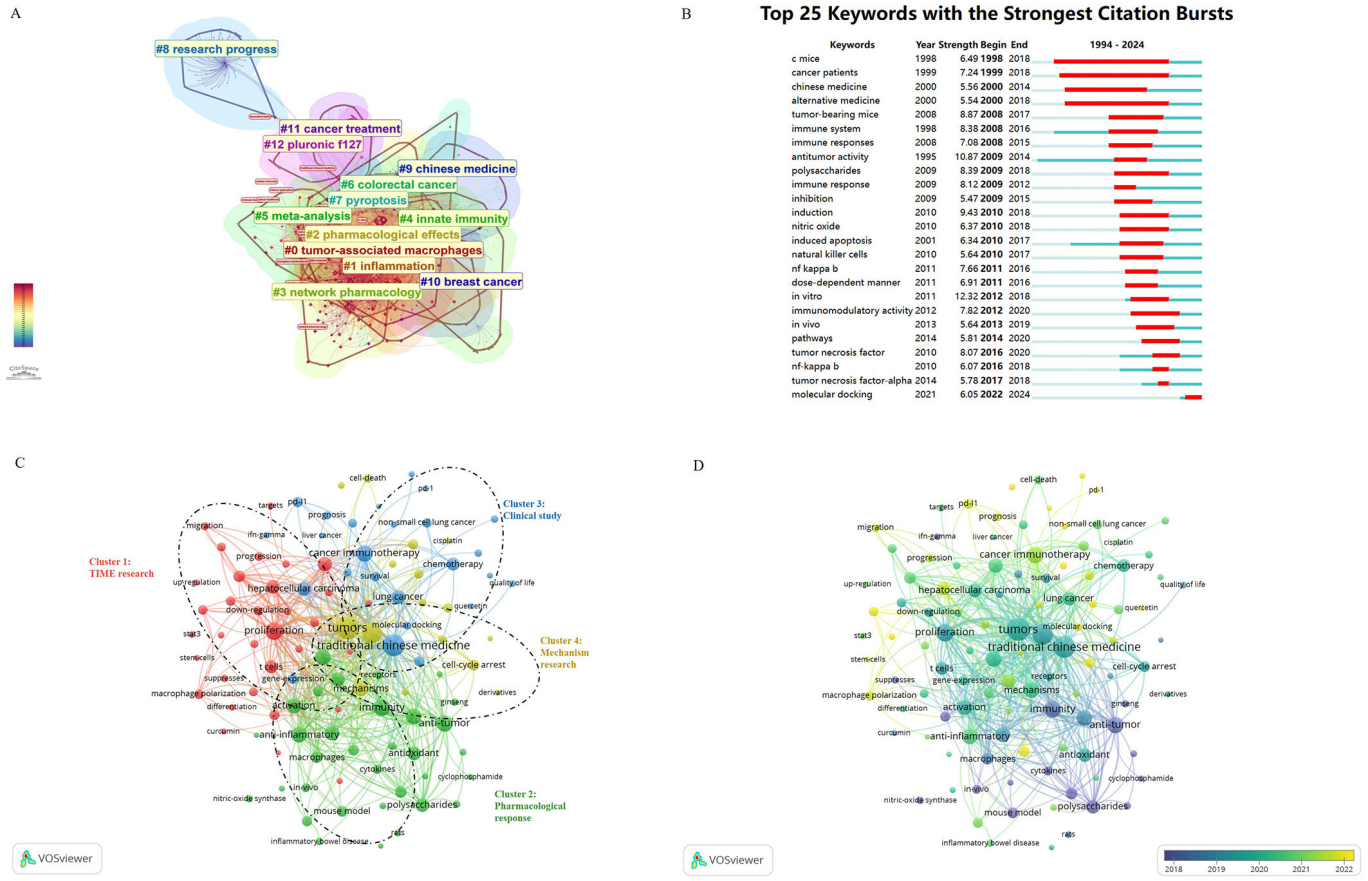
Visualization of co-occurrence keywords analysis based on TCM in cancer immunotherapy research. **(A)** Clustering analysis of the co-occurrence keywords network based on CiteSpace. (Modularity Q = 0.4377, Weighted Mean Silhouette S = 0.7277) **(B)** The keywords with the strong citation bursts in articles related to this field. **(C)** Mapping of keywords in the research on TCM in cancer immunotherapy. The keywords of research fields were divided into four clusters by different colors, and the frequency is represented by point size. (Items:107; clusters:4; links:3364; Total link strength:16232) **(D)** Distribution of keywords according to their time of appearance. The yellow color represents an earlier appearance and blue point appeared later. Created with VOSviewer.

The keywords in the publications accurately reflected active research topics. To reveal current research trends in TCM in cancer immunotherapy, 258 keywords were gathered from publications, and a network map was created to visualize keyword clusters. Accordingly, “tumor-associated macrophages” (Cluster 0), “inflammation” (Cluster 1), “pharmacological effects” (Cluster 2), “network pharmacology” (Cluster 3), “innate immunity” (Cluster 4), “meta-analysis” (Cluster 5), “colorectal cancer” (Cluster 6), “pyroptosis” (Cluster 7), “research progress” (Cluster 8), “Chinese medicine” (Cluster 9), “breast cancer”(Cluster 10), “cancer treatment” (Cluster 11), and “pluronic F127” (Cluster 12) have been research hotspots since 1994 (**Figure 8A**).

Identifying ‘burst keywords’ could help predict future study trends and emerging areas of interest. The CiteSpace algorithm was also used to detect keyword bursts based on burst detection analysis. As presented in **Figure 8B**, the keywords ‘mice’ (burst duration from 1998 to 2018), ‘cancer patients’ (burst duration from 1999 to 2018), and ‘alternative medicine’ (burst duration from 2020 to 2018) have received the most consistent attention over time. TCM was an important part of complementary alternative medicine. In addition to being an alternative medicine in the form of herbs, herbal extracts, compound preparations, etc., TCM could also be made into nanomedicines, which were involving in the modulation of tumor immunosurveillance to enhance antitumor activity. These results also showed that the development of the novel dosage form of TCM for anti-tumor immunity was a very important research direction. Interestingly, the result was consistent with the keyword clustering results shown in **Figure 8A**. For instance, “pluronic F127” (Cluster 12) was known as a surfactant used in drug delivery systems, after coating immune adjuvant, nanomedicines were obtained, which maintained high stability during their passage through the gastrointestinal tract, achieving efficient colonic mucus infiltration and specific tumor penetration. In addition, keywords such as ‘molecular docking’ (burst duration from 2022 to 2024), ‘tumor necrosis factor’ (2016-2020), ‘nf-kappa b’ (2016-2018), and ‘immunomodulatory activity’ (2012-2020), have gained traction more recently. As seen from the burst keyword chart (**Figure 8B**), we can get a general idea of how themes are evolving. For instance, the research trend of TCM tumor immunotherapy has changed from observing the changes of immune indexes in patients and model animals at the very beginning to exploring the mechanism of TCM tumor immunotherapy in more and more studies. In recent years, researchers have begun to dig deeper into the pharmacodynamic molecules of anti-tumor immunity. All these lay the foundation for the development of precision targeted drugs. The increasing interest in research on molecular mechanisms and potentially effective components of TCM in cancer immunotherapy has probably opened new research frontiers in this field.

Keyword co-occurrence analysis was a prevalent method for investigating popular research topics and areas, and also played an important role in surveying the developments of scientific research. Keywords, defined as terms used ≥ 15 times in the titles or abstracts of all papers, were included in the study. The 107 keywords identified were divided into four clusters according to their relevance. As shown in **Figure 8C**, the red cluster was related to the “tumor immune microenvironment,” with keywords such as “tumor-associated macrophage,” “tumor microenvironment,” “epithelial-mesenchymal transition,” and “astragalus polysaccharides,” indicating the pharmacological action of TCM in anti-tumor immunity. Cluster 2, shown in green, focused on “pharmacological response,” with keywords such as “flavonoids,” “pharmacology,” “anti-inflammatory,” and “immunomodulatory,” indicating modes of interaction between the TME and tumor. The blue cluster was related to “clinical research,” focusing on the clinical efficacy of conventional treatments, with keywords such as “biomarkers,” “chemotherapy,” “immune checkpoint,” “cancer immunotherapy,” “PD-L1/PD-1,” and “prognosis.” The yellow cluster was mainly related to the “anti-tumor mechanism of TCM regulating immunity,” with keywords such as “ferroptosis,” “immunogenic cell-death,” “autophagy,” “cell-cycle arrest,” and “mitochondria”. These results showed that the most prominent applications of TCM in cancer immunotherapy include the aforementioned four directions. TCM in cancer immunotherapy can be based on the type of tumor and the patient’s physique to develop a personalized treatment plan to improve the therapeutic effect. However, in general, the study of TCM in tumor immunotherapy is not deep enough, there is a lack of large-scale clinical research evidence, and the evaluation criteria of efficacy are not clear. Although we have made some achievements in the study of pharmacological action and molecular mechanism, the study of side effects, toxicity and modern preparation of TCM is insufficient.

Additionally, these co-occurrence keywords were coded with different colors by VOSviewer based on their average frequency of appearance in all published papers (**Figure 8D**). Blue indicated keywords that appeared earlier, whereas yellow indicated those that appeared later. Early research primarily focused on “anti-tumor”, “immunity”, “ginseng”, “polysaccharides”, “cytokines”, and “mouse-model”. On the contrary, topics related to “PD-1/PD-L1”, “prognosis”, “quercetin”, “molecular docking”, and “macrophage polarization” gained more attention in recent years. These results indicated that the use of TCM in cancer immunotherapy has gradually shifted from *in vivo* and *in vitro* models to clinical efficacy. Simultaneously, the research focus has changed from compound TCM preparations or classes of ingredients to specific pharmacodynamic ingredients, with corresponding targets transitioning from cytokines to immune checkpoints.

## 4 Discussion

**Figure 9:**
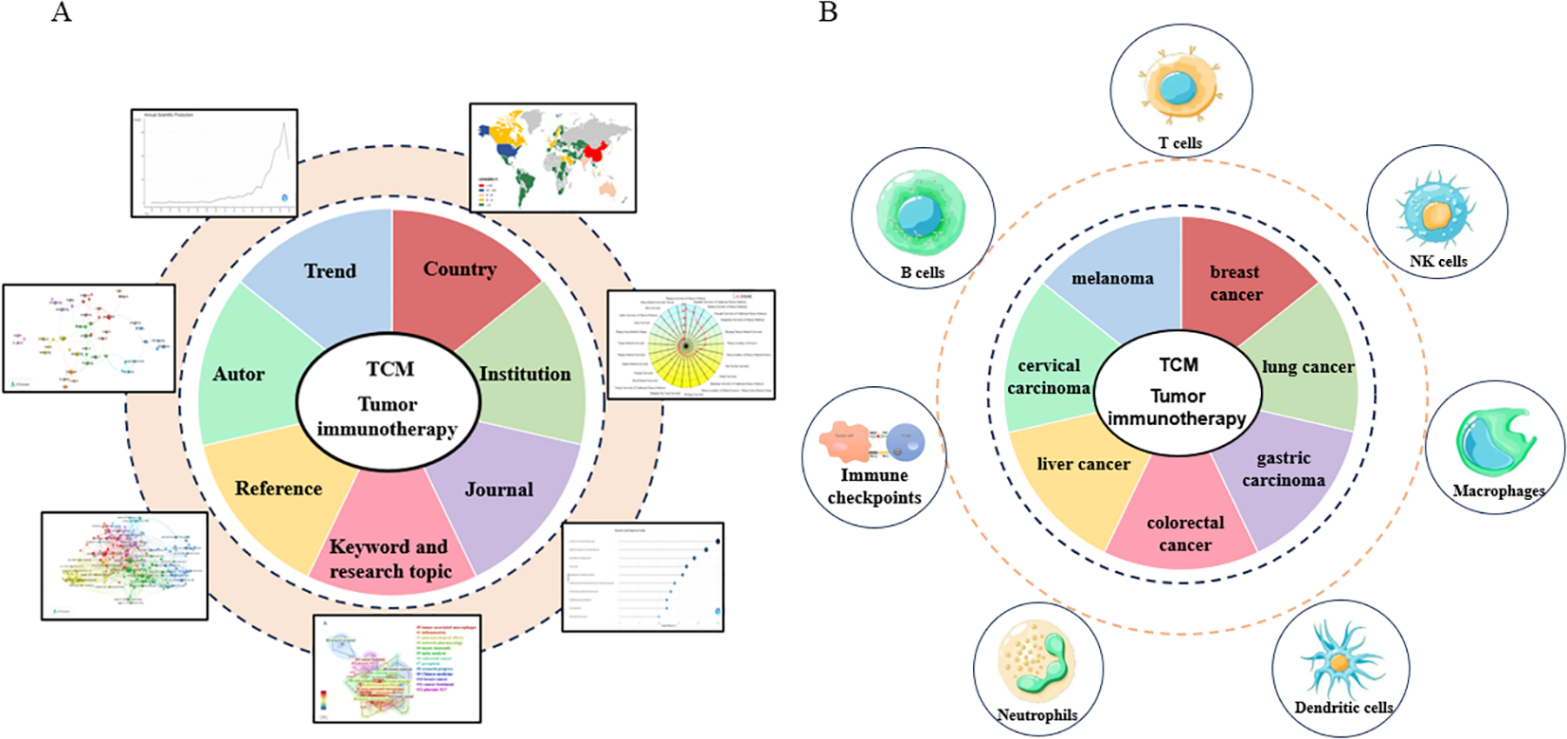
**(A)** Flow chart of research approach of this study. **(B)** Tumor types and targeted immune system of TCM for tumor immunotherapy.

Cancer is induced by genetic mutations and the disruption of cellular homeostasis. The extracellular environment resulting from these changes is known as the TME, which contains various immune and immunosuppressive cells, including NK cells, CD8/CD4 T cells, M1 and M2 macrophages, Tregs, and MDSCs. These cells mediate immune regulation by enhancing the tumor immune response or promoting cancer cells’ evasion of immune surveillance. Therefore, tumor immunotherapy aims to enhance the immune response by activating immune cells, particularly by eliminating residual tumor lesions, inhibiting tumor growth, and disrupting tumor immune tolerance. TCM is primarily used as an adjuvant therapy in cancer treatment and regulates overall immune function. Researchers have found that TCM encourages immune regulation and plays a crucial role in immunosuppression, affecting various immune cells and cytokines in the TME (28). Research on the application of TCM in cancer immunotherapy has also made breakthroughs that could provide novel concepts for the development of new anti-tumor drugs. With icaritin, the world’s first-in-class small molecule anti-hepatocellular carcinoma immunomodulatory drug independently developed by China, receiving approval from regulatory authorities, research on the anti-tumor immunity of TCM has received greater attention. To further investigate and describe the current status and future directions of TCM in cancer immunotherapy, we analyzed publications in the field from 1994 to 2024 using bibliometric analysis methods, thereby paving the way to understanding research progress and development trends in this field (**Figure 9**).

### 4.1 General information

Our analysis revealed a notable upward trajectory in the publication of research related to TCM in cancer immunotherapy over the past 3 decades, with a marked increase in the last 5 years. This trend suggested a growing interest and focus among researchers in this field. However, the low publication numbers observed in 2024 can be attributed to the timing of the data export, which was conducted in April of that year. Overall, these insights underscored the increasing relevance of TCM in cancer immunotherapy and suggest that it is an area of study warranting further exploration and investment. According to the global publication trend forecast, the number of publications in this field might continue to rise in the future.

### 4.2 Status of authors

**Figure 5B** showed the clustering network diagram with authors such as Wang Yitao as the center node, indicating that these authors were in a leading position in this field. In addition, combined with the author’s production over time (**Figure 5A**), we found that Wang Yitao’s articles have outstanding advantages in citation. After digging deeply into the research content of Professor Wang Yitao’s team, we found that the team has achieved fruitful results in the research of TCM anti-tumor immunity. It includes the mechanism research (29), the development of new immune active substances of TCM (30), the research of new targeted drug delivery system (31) and the application of multi-omics research methods (32). The team provided us with theoretical and technical guidance from multiple research directions of TCM anti-tumor immunity, and the research methods and research ideas are worthy of our reference.

### 4.3 Geographic distribution and culture features

About the geographic distribution of research outputs, the research indicated that the highest pooled prevalence of the usage of herbal medicine in adults with cancer was in Africa (47%), followed by Asia (30%), America (21%) and European (18%). From an economic viewpoint, low- and middle-income countries (33%) were more likely to have a high prevalence of herbal medicine use by adults with cancer than high-income countries (19%) (33). It may due to socio-economic reasons, many African people have resorted to traditional medicine using natural products to treat diseases, including cancer (34). While this study showed that, except for China and India, the America and Germany have a higher centrality on research collaborations. This is contrary to the prevalence of herbal medicine usage, largely due to the insufficient economic support for scientific research in African countries.

Some studies about cancer with complementary and alternative medicine showed that, Japan (35) and Thailand (34) maintain a positive attitude towards the integrative cancer therapy, this may be related to cultural, national policies and other factors. For example, in 2013, President Xi Jinping launched the Belt and Road Initiative, and the Chinese government has identified TCM as an important health commodity for the global market. TCM was also promoted as cultural power along the Southeast Asian belt and road corridor (36).

In additional to TCM, as a complementary and alternative medicine (CAM) for cancer immunotherapy, there are Ayurvedic medicine, European medieval medicine, Kampo medicine worldwide. The researches about European medieval medicine are much less than other CAM practices (37). Ayurveda was originated and evolved in India and had been practiced for thousands of years, and Curcumin, Ashwagandha, and Triphala were three of the most promising and commonly researched Ayurveda herbs or formulas related to cancer. Ayurvedic medicine foucused on ‘one target-one herb’, and the researchers has realized this approach’s limitation (38). Kampo medicine (Japanese traditional medicine) was originated from ancient China, and had been improved since the Edo period. Most Kampo preparations were available as extract formulations, which was greatly different from China, and only 148 formulations were covered by health insurance system (39). Compared with China, there were 2282 kinds of TCM collected by the 2023 edition of the “China Medical Insurance Directory” (40). The current researches indicated that TCM was used as adjuvant therapy to treat dozens of cancers efficiently, and they exhibited prospective antitumor potential with multiple targets, multiple signaling pathways and less side effects (41, 42). Moreover, the Chinese government also strongly supports the development of TCM through national policies such as the Belt and Road Initiative and the Forum on China-Africa Cooperation (43, 44). Under these policies, China aims to construct a TCM international medical service system, so it will also make the distinctive contributions to the field of cancer immunotherapy.

### 4.4 Hotspots and development tendency

The first hotspot and trend in TCM for cancer immunotherapy were cancer types. Compound TCM preparations used either alone or as adjunct therapies, have been widely used in cancer treatment (45). Keyword co-occurrence analysis revealed that “lung cancer”, “liver cancer”, “colorectal cancer”, “breast cancer”, “gastric cancer”, “cervical carcinoma” and “melanoma” were the most researched cancer types in this field. For example, Tang demonstrated that Jin-Fu-An decoction targets lung cancer by modulating macrophage polarization via β-catenin synergy (46). Chen et al. found that Dahuang Zhechong pills enhanced anti-tumor immunity in liver cancer by secreting IFN-γ to activate CD8^+^ T cells and promote Treg differentiation (47). Jiang et al. showed that Tong-Xie-Yao-Fang inhibits the growth of colorectal tumors by promoting the maturation of dendritic cells and stimulating T cell-mediated immune responses (48). XIAOPI was confirmed to inhibit breast cancer lung metastasis by inhibiting CXCL1 secretion from tumor-associated macrophages (49). Bu-zhong-yi-qi decoction modulates peripheral immunity and suppresses the immune escape of tumors via PD-1/PD- L1-dependent T cell immunization, showing promise for gastric cancer therapy (50). Additionally, Zhang et al. revealed that flavonoid extracts from *P. heterophylla* have anti-tumor and immunomodulatory effects in cervical carcinoma-bearing mice by activating more CD4^+^ and CD8^+^ T lymphocytes (51). Furthermore, triptolide significantly inhibited tumor growth by stimulating antigen presentation of dendritic cells (DCs) and the proliferation of cytotoxic T lymphocytes (52). In general, there were fewer studies on tumors of the upper respiratory tract, cervical cancer, and melanoma. It may be related to the relatively low incidence of cancers mentioned above. According to the Global Cancer Statistics 2022, lung cancer is the most commonly diagnosed cancer worldwide (12.4% of the total cases), followed by cancers of the female breast (11.6%), colorectum (9.6%), prostate (7.3%), and stomach (4.9%) (53). In addition, cancers such as melanoma, which have a high degree of malignancy, rapid disease progression, and a short course of disease, may not be very suitable for the use of TCM as an alternative therapy.

The second hotspot and trend in TCM related to cancer immunotherapy was the evolution of research methodologies. Despite limited research and support for the effectiveness of TCM, it has gained significant attention in recent years, prompting more scientists to employ scientific methods to elucidate its fundamental mechanisms, safety, and efficacy. Existing research methods included network pharmacology, bioinformatics, multi-omics analysis, meta-analysis, *in vitro* and *in vivo* experiments, and clinical trials (54–58). By combining the overlay visualization map of keyword co-occurrence and the burst chart, we found that molecular docking technology has recently become a widely used method. Since its inception in the mid-1970s, molecular docking has proven to be an essential tool for efficiently understanding the interactions between chemical compounds and their molecular targets, and for drug discovery and development (59). The ambiguity of active ingredients and therapeutic targets remained a limiting factor for the broader clinical application of TCM in cancer immunotherapy. Keyword co-occurrence analysis showed that research on TCM anti-tumor immunity has gradually shifted from *in vivo* and *in vitro* experimental studies to the development of clinically targeted drugs. Additionally, research has increasingly expanded from compound TCM preparations to individual pharmacological ingredients (58, 60). Therefore, molecular docking combined with multi-omics analysis was a popular and trending research method for TCM in cancer immunotherapy. For example, techniques of network pharmacology, molecular docking, and experimental validation were applied by Lu to demonstrate the mechanism of BZD for anti-CRC. As a result, quercetin, kaempferol, licochalcone A, naringenin, and formaronetin were more highly predictive components related to the T cell activation in colorectal cancer mice by Molecular docking and experimental validation (54). Gao determined the top 10 key proteins in the upregulated KEGG pathways of PMN-MDSCs in melanoma tumour-bearing mice through proteomics and Cytoscape analysis. Then PMN-MDSC inhibitor prim-O-glucosylcimifugin was screened by molecular docking from the TCM Library. Finally, the effect of the inhibitor was verified through proteomics and metabolomics analysis in melanoma and triple-negative breast cancer mouse tumour models (61). Nevertheless, the results of molecular docking are often accompanied by significant uncertainty, which is easy to produce false positive results. Next, molecular docking usually relies on server resources, which requires high computing resources, and the calculation process is time-consuming. In addition, the technology requires high quality of input data, requiring that the input protein structure needs to undergo strict pretreatment, including structural repair, hydroprotolation, etc., to ensure the accuracy of docking. In summary, although molecular docking technology has some shortcomings, its ability to efficiently predict binding patterns and affinity makes it of great value in the field of drug design and biological research. By combining the experimental data and the optimized calculation method, the accuracy and reliability can be further improved (62).

The third hotspot and trend in TCM for cancer immunotherapy involved molecular mechanisms. Anti-tumor immune responses could be initiated through innate and adaptive immune systems. TCM has also been reported to exert immunoregulatory effects by upregulating immune responses, particularly in the TME. Studies have shown that TCM exerts its immunomodulatory function by regulating the differentiation and cytokine secretion of macrophages, dendritic cells, NK cells, MDSCs, T cells, and B cells, thereby enhancing the ability of immune cells to kill malignant cells or present antigens (48, 63–66). Currently, there were few clinical studies on TCM affecting immune checkpoints. Keyword time-zone map analysis showed that recent studies have focused on the inhibitory effects of TCM on immune checkpoints. Researches showed that TCM could directly reduce PD-1 and PDL-1 expression in tumor cells. In addition, TCM could remodel the gut microbiota to block PD-1 expression in cancer (67, 68).

Fourth, to clarify the timing of TCM use as an immunomodulatory agent, we included reports of cancers and inflammatory diseases highly associated with cancer, such as chronic liver disease and ulcerative colitis. Wang conducted a meta-analysis concluding that patients with stage III/IV non-small cell lung cancer (NSCLC) could safely receive elemene injections alongside platinum-based chemotherapy, which enhanced clinical efficacy, improved cellular immune function, and reduced chemotherapy toxicity (69). Relevant clinical investigations have shown that combining Qing Gong Detoxification Soup with TP (paclitaxel-cisplatin) chemotherapy significantly restored immune homeostasis, inhibited tumor markers, and reduced the incidence of adverse reactions in patients with cervical cancer after surgery (70). Chronic HBV infection could ultimately lead to liver cirrhosis and hepatocellular carcinoma, which were major complications and leading causes of mortality worldwide (2). Reports indicated that curcumin’s antiviral activity, which targeted and modified regulatory T cells into T helper cells, could be enhanced by blue laser photobiomodulation, facilitating the elimination of HBV-infected hepatocytes (71). According to the results of keyword co-occurrence analysis, “inflammatory bowel disease” (IBD) was the most studied inflammatory disease at present. Natural products and herbal medicines have demonstrated efficacy for IBD in experimental models and clinical trials by modulating innate and adaptive immune responses, regulating macrophage activation, and inhibiting TNF-α activity (72). In summary, immunotherapy using TCM could contribute to effective maintenance or adjuvant therapy throughout the whole course of cancer instead of only in the late stages, enabling more patients to live with cancer for longer periods.

### 4.5 Strengths and limitations

We conducted a systematic visualization analysis of the articles and trends in TCM for cancer immunotherapy in an intuitive way for the first time, providing new research directions for scholars in this field. However, our research also had some limitations. For example, due to limitations in the current mainstream bibliometric software, the data from different database cannot be integrated, such as PubMed, CNKI, and patent information in both Chinese and English. Therefore, we will use more Chinese database solely to supply this research and more powerful software are supposed to be used in the future. In addition, the input data was extracted using carefully designed search strings, but some irrelevant articles were still retrieved, and relevant publications with unclear titles might have been missed. Furthermore, the literature analysis software used (CiteSpace, VOSviewer, and Bibliometrix Package) might produce slightly different statistical results due to version discrepancies. As with any statistical endeavor, bibliometric analyses have the potential to generate misleading and biased results. Finally, recently published high-quality articles might not have received attention because of their temporarily low citation counts. Despite these limitations, our study provided insights into the characteristics of research and citations in the field of TCM for cancer immunotherapy.

## 5 Conclusion

Our research was the first to provide a scientific and comprehensive overview of the research trends in TCM in cancer immunotherapy over the past 30 years. These findings systematically summarized global research trends to help scholars navigate this domain more effectively and pursue innovative directions in their studies. With the development of AI technology, the emerging interdisciplinary field of TCM Network Pharmacology (TCM-NP) will combine AI methods and multi-omics data to provide a clearer basis for further understanding of the theory and mechanism of TCM in cancer immunotherapy. In addition, we should accelerate the organic integration of TCM and Western medicine in the future, and promote the modernization of TCM from clinical practice, fundamental research, technology application, and standardization construction. Driven by China’s Belt and Road Initiative and other government policies, we expected that further cooperation among authors, institutions, and countries in the future would help researchers conduct more in-depth and systematic research to provide accurate guidance for clinical medication, as well as promote the internationalization of TCM, and enhance its international status.

## Data Availability

The original contributions presented in the study are included in the article/supplementary material. Further inquiries can be directed to the corresponding authors.
